# High resolution descriptors for UAV mapping in biodiversity conservation – A case study of sandy steppe habitat renewal

**DOI:** 10.1371/journal.pone.0315399

**Published:** 2025-03-13

**Authors:** Maja Arok, Branko Brkljač, Predrag Lugonja, Bojana Ivošević, Milan Vukotić, Tijana Nikolić Lugonja

**Affiliations:** 1 BioSense Institute - Research and Development Institute for Information Technologies in Biosystems, University of Novi Sad, Novi Sad, Republic of Serbia; 2 Department of Power, Electronic and Telecommunication Engineering, Faculty of Technical Sciences, University of Novi Sad, Novi Sad, Republic of Serbia; 3 Ranger service, Public enterprise Palić-Ludaš, Palić, Republic of Serbia; Jinan University, CHINA

## Abstract

Due to the large-scale disappearance of grasslands there is an urgent need for revitalization. It calls for consistent and accessible monitoring and mapping plans, and an integrated management approach. However, revitalization efforts often focus solely on the vegetation component, and skip the link to other animal species that perform vital functions as ecosystem engineers and umbrella species. In this study, we combine an in-situ standard phytocoenological survey with an UAV-based technology in the effort to improve the monitoring and mapping of the sandy steppe habitat of the European ground squirrel (*Spermophilus citellus*; EGS), undergoing revitalization in the northern Serbia. It is a model organism of an animal species that enables identifying habitat quality and quantity indicators to understand the broader implications of the ecosystem revitalization efforts on the wildlife populations. The proposed approach tested whether the commercially available RGB sensor and a relatively high flight height of the UAV have discriminative capacity to aid site managers by mapping identified steppe development stages (specific plant assemblages, reflecting different habitat types). Thus, a novel set of high-resolution image descriptors that are capable of discriminating plant mixtures corresponding to Fallow land, Forest steppe and shrubs, Young steppe I and II, was proposed. Despite high resolution imaging, the method solves a challenging problem of UAV vegetation mapping in the case of limited spectral and spatial information in the image (by using only RGB camera and multitemporal approach). Although the lack of visual information that would allow identification of individual plant parts and shapes prevented the use of usual object-based image analysis, proposed pixel-based descriptors and feature selection were able to provide the extent of the targeted areas and their compositional carriers. Presented holistic approach enables implementation of effective management strategies that support the entire ecological community.

## Introduction

One of the strategic goals for preserving natural diversity is the restoration of degraded natural habitats [1,2]. Restoring it in the framework of the integrative approach implies habitat management and monitoring linkages [3]. In other words, setting conservation priorities for the restored habitat with a logical set of categories – condition indicators and limits of restored habitat development phases is essential. Studies assessing restored habitat quantity (range and area) and quality (structure, function, and typical species) include the use of various methods and tools [4–7]. For example, plant species diversity monitoring in restored habitats is common practice for further management and development of conservation strategies. However, animal species’ critical role in tracking habitat restoration is rarely considered [8].

The recent decade’s development of remote sensing (RS) technologies has significantly impacted habitat detection and characterization [9,10]. Besides satellite coverage of land and habitat types, Unmanned Aerial Vehicles (UAVs) equipped with different types of sensors have brought additional quality to the information we collect about the various habitat classes [11–13]. Furthermore, high-resolution UAV optical imaging has enabled the mapping of details at the level that requires developing novel image analysis techniques and processing approaches for extracting valuable insights into grassland ecosystem monitoring [7]. Grassland biomass estimation, predicting foraging quality, and mapping invasive species in grasslands are all applications of UAV-born RS [14–16]. Furthermore, revitalized grassland habitats assume mapping of the spatial distribution of vegetation development phases, which is essential for determining indicators of habitat quality. Thus, developing approaches for local habitat quantification based on UAV image attributes is necessary.

There have been efforts to detect and characterize habitats using remote sensing and modeling tools to inform their distribution, conservation status, and local management plans at various spatial resolutions and scales, e.g. [17–19]. But the tools to assess restored habitat quality to inform local management conservation plans still need to be improved. Moreover, if available, such tools can, e.g., improve the management of revitalizing grassland habitats that vanish due to intensive agricultural development or land abandonment. These habitats are home to endemic and endangered plant and animal species, some of which perform vital functions [20–26]. Therefore, their joint monitoring in any restored habitat assessments is essential. In that sense, one of Europe’s most endangered open grassland habitats today is the steppe, which represents only remnants of the once vast open grassland [27–29]. Thus, policies like Annex I of the Habitat Directive [30] recognize steppe habitats as endangered. Furthermore, an important historical and ecological element of European steppe habitats is the endemic, endangered small mammal – the European ground squirrel (*Spermophilus citellus*, EGS). Being centrally positioned in the trophic web, the EGS serves to connect different trophic levels. On the one hand, it feeds upon the locally distributed grassland plant species, while, on the other, it represents an important prey for predators, including birds and mammals [31–35]. Lastly, because it is a habitat specialist, the EGS can be used as a model species for assessing semi-natural grassland habitats’ conservation potential.

Combining multitemporal measurements with spatial characteristics of vegetation is a standard mapping approach for imaging grassland areas when no color information is available [36]. In such cases, statistical features (e.g., image moments or entropy) are computed for a predefined window size around each pixel in the scene to capture the spatial information about its texture. However, as pointed out by [37], image characteristics that define the habitat type are often related to fine-scale details in complex spatial arrangements and variability in space and time. In that sense, pixels’ surroundings can be considered fine-scale objects that are hard to describe explicitly but implicitly capture the abstract features that characterize the habitat type or some specific entities. It is especially true in classification problems where individual plants are not visible or multispectral measurements are unavailable (measurements do not allow finer spectral characterization of plants or their mixtures). In such cases, image texture and multitemporal observations provide the most discriminative information about classification categories. It also holds for medium to high-resolution satellite images, which offer broader area coverage compared to UAV platforms but can be inappropriate for mapping grassland habitat types characterized by plant mixtures at centimeter scales. Classification of the grassland successional stages can also be tackled by airborne hyperspectral imaging [38]. Even in such cases, where most information is in the spectral domain, a small window around each 1x1 m pixel is usually used for noise-canceling spectral signatures by spatial averaging [38]. Similarly, in the case of high-resolution multispectral satellite imaging at 30 cm [39], classification is also performed by spatial aggregation of information over the 3x3 m local neighborhood.

**Fig 1:**
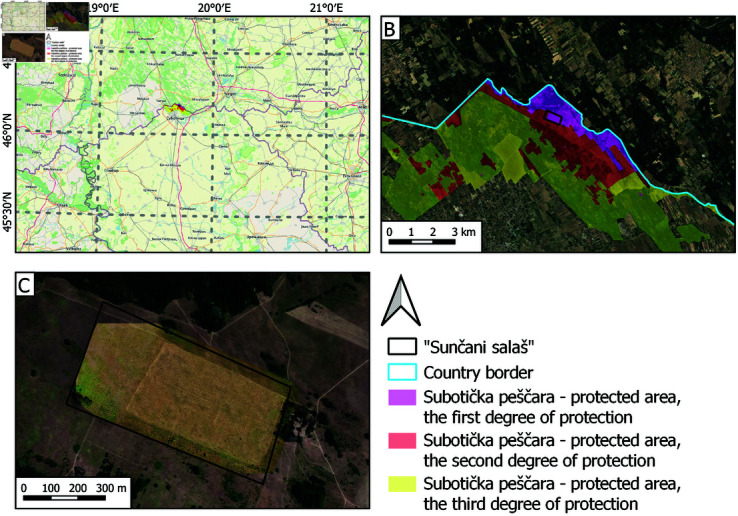
Location of the study area. A: Landscape of outstanding features Subotička peščara. B: Satellite image of the sandy steppe habitat Sunčani salaš, C: overlapped with an UAV orthophoto mosaic from 09/30/2021.

Mapping experiments in this study (Fig 1) investigated the potential of UAV optical imaging in providing reliable grassland land cover maps under challenging operational conditions. The assumptions are that the land managers mostly have access only to low-cost RGB sensors and that the UAV acquisitions are taken from a relatively high flight height to achieve broader spatial coverage in a single flight mission. Under such circumstances, determining image areas corresponding to different habitat types, i.e., plant mixtures corresponding to sets of relevant plant indicator species identified by domain experts, can be challenging due to lack of spectral and spatial details. For example, the presence of varieties is characteristic of certain steppe development phases and is particularly interesting for monitoring the habitat renewal process. Although commercial RGB cameras do not provide fine spectral characterization, such wideband sensors are highly affordable and easy to deploy.

The lack of spatial details at higher UAV flight heights prevents visual identification of individual plants or their specific parts in the scene. Therefore, conducted UAV mapping experiments can be regarded as pixel-based classification approaches similar to the ones described by [39], and [40], which are exploring discriminative information contained in the texture features and the pixel’s local neighbourhood. Somewhat different approaches combining satellite imaging and UAV data have been described by [41], while combining hyperspectral measurements and image textures was considered in [42]. In that sense, local image features that implicitly represent objects of interest [37] can be utilized as sources of discriminative information, as recently also demonstrated in [43], where pixel-based classification approaches have been utilized to detect burrows with only a few centimeters in size.

In order to capture the structural information about the plant mixtures that are characterizing each of identified habitat types, we have decided to rely on multiscale texture features, like gray-level co-occurrence matrices (GLCMs) and morphological profiles (MPs), which reflect the higher order spatial statistics around each sampling point. Due to relatively limited number of ground-truth samples (vegetation sampling plots continuously monitored over the growing season) it was not possible to pursue an alternative data-driven approach for design of problem specific high resolution image descriptors based on contemporary deep learning (DL) techniques that rely on large training sets. Thus, instead of DL methods [44,45], which have been utilized in similar UAV mapping tasks, an ensemble type classifier in the form of random forest was utilized in combination with hierarchical feature selection in order to identify feature importance and perform image classification. Besides the training set size, the presence or lack of discriminative visual features, i.e. relationship between the UAV mapping ground sampling distance (GSD) and plant size, plays crucial role in the design choice of high-resolution image descriptors and corresponding image classifiers. In [45] it was shown that at already 2 cm GSD per pixel, the DL model performance can be severely degraded in the case of detection of individual plants. On the other hand, the mapping problem in our study is dealing with complex plant mixtures consisting of varying amounts of several plant species at the given test site (Fig 1), i.e. relying mostly on spatial information about the classification categories of interest, as compared to DL object detection approaches proposed in [44,45], or [46], where DL is used to aggregate rich spectral information over small image patches. In that sense the proposed method is more similar to DL approaches described in [47,48], where spatial characteristics of whole vegetation instead of individual plants were captured by UAV. However, the main difference comes form the level or scale at which the vegetation characterization was performed during the ground-truth sampling. The vegetation sampling (characterization of plant mixtures) in our study was very detailed and limited to small 1 m x 1 m sampling plots, which did not allow for object-based approaches or classification of small image patches using DL due to limited number of training samples.

In order to capture the phenology of different plant species and compensate for missing information in spatial and spectral domains, time series of UAV images were always considered as the input data for classification experiments in this paper. However, only as indicators of seasonality effects, without detailed analysis of phenological stages, like the start and end of the growing season [49].

In general, the optimal number of measurements depends on the specific mapping target, environmental conditions, and inter-annual variability of phenological development [50], and it should be determined for each application separately. Experiments by [51] suggested that the early summer acquisitions should be the most informative ones. In semi-arid landscapes, single-date images may also provide a reliable method for thematic mapping [52]. However, dense time series are considered as better choices for small-scale vegetation typical for grassland habitats [50], revealing the evolution of greenness per area and different blossoming phases. Other temporal characteristics include vegetation height and structure changes captured by digital surface models and spatial image descriptors. However, such clues can be affected by the hay cut and similar mowing land management operations.

In the context of revitalized steppe habitat and survival of EGS species, we performed our experiment on the selected test site, *Sunčani salaš*, Fig 1, where along with promoted grassland revitalization the renewal of the European ground squirrel local population is taking place. By identifying the habitat quality and quantity indicators through a combination of phytocoenology and remote sensing methods, we could define specific characteristics of steppe development phases and provide valuable insights into the level of habitat renewal at the test site. These insights were the basis for design of automated image classification that goes beyond human image interpretation, with maps denoting spatial regions at different steppe development phases, i.e., location and extent of management zones of interest.

**Fig 2:**
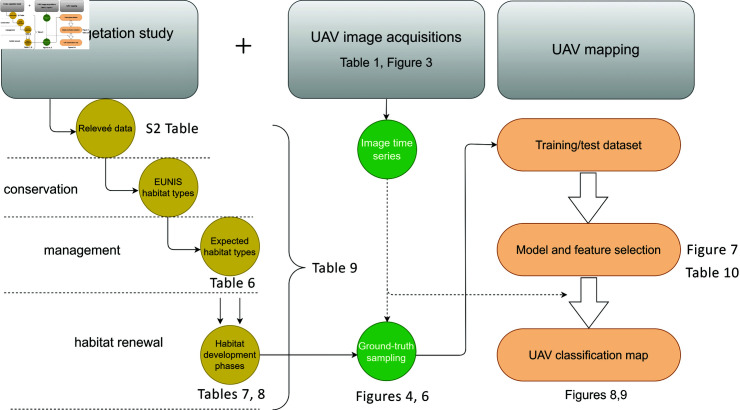
An overview of research workflow. All activities were grouped around three main topics shown in the upper part of the diagram. Next to each functional block are the names of the corresponding Tables and Figures in the text. E.g. Tables 8 and 9 summarize the main results of the proposed vegetation study and the performances of the designed UAV time series classification models.

The main goals of the presented study were to:

1. Establish methodologies for continuous monitoring of revitalized steppe, by considering a) habitat quantity (extent of steppe development phases); and b) habitat quality (e.g. compositional characteristics of development phases, presence of alien species, etc.);
2. Investigate the potential of automated UAV mapping of identified steppe development phases under the assumption of RGB images taken at high flight height; and
3. Identify the conservation status of development phases of revitalized steppe habitat – summarized in Fig 2, which depicts the overall research workflow.

Finally, the research aimed to support the development of a management program for a small herbivorous mammal, the European ground squirrel. The availability of an automated mapping system and accompanying spatial analysis tools is therefore of great importance in reducing the overall cost of long-term operations and improving the pace of renewal. Thus, establishing such systems will improve habitat management and the long-term viability of the EGS population.

## Materials and methods

### Study species

Range of the European ground squirrel (EGS, *Spermophilus citellus* L. 1766 Rodentia: Sciuridae), a globally endangered and strictly protected species in Serbia [53,54], has decreased by 70% in the Vojvodina region (northern Serbia) over the past 70 years [55]. The species is considered an open grassland habitat specialist, i.e., it avoids sites covered with high vegetation and agricultural parcels [56]. Following a dramatic decline in grassland coverage across Europe, the EGS lost most of its habitats. Across Europe, the species’ distribution today is highly fragmented, and many populations are isolated. In Serbia, most of the remaining populations reside in the northern Serbian province of Vojvodina, inhabiting patches of steppe and saline habitat in the predominantly agricultural area. In the agricultural matrix of northern Vojvodina, Fig 1A, the remaining small number of EGS colonies are scattered through parcels covered with alfalfa (*Medicago sativa*). When alfalfa is replaced with another grain in crop rotation (which usually involves deep plowing), animals in underground burrows become directly endangered. Furthermore, alfalfa is generally followed by corn, wheat, and other cultures, which, because of their height, form “impenetrable” surfaces for the EGS, thus preventing the movement of animals between colonies and further reducing the gene flow.

### Study area – “Sunčani salaš” sandy steppe habitat

Sunčani salaš, Fig 1, is located within the Landscape of outstanding features Subotička peščara, a protected area of national importance [57]. It was selected as a suitable test site for the described habitat renewal process and EGS reintroduction to the area. The site covers approximately 30 ha split between protection category zones I and II, Fig 1. Since 2020, the site is a part of the LTER (Long-Term Ecosystem Research) network: https://deims.org/5f5c850d-0036-49ac-97be-f9b314898607.

The revitalization of the steppe habitat, formerly used as arable land, began in 2010 after the regional and local government extended the protected area and acquired the parcel from private ownership. The conservation priority of the area managing body (Palić-Ludaš public enterprise, http://www.palic-ludas.rs/index/page/id/97/lg/sr) was the revitalization of the habitat that will support an EGS colony. The revitalization was primarily passive, with very little active management. In 2018, the Provincial Institute for Nature Protection’s professional service rated the regeneration a success. In the same year, the area management body established management consisting of alternating mowing (early summer) and grazing (200 sheep) at the site. Once the habitat is satisfactory for EGS, the activities to help the subsequent adaption of the individuals and maintain a viable EGS population occur.

### Renewal goals

Sandy steppe habitat renewal through grassland revitalization efforts at the selected test site represents the first necessary step for reintroducing EGS to its historic habitat. Furthermore, establishing a functional EGS population at the Sunčani salaš site is only the initial step towards repopulating the wider grassland area of the Subotica sands. In the long term, translocations of individuals from the existing agricultural fields to their former natural habitats should enable the formation of a meta-population structure in which agricultural areas serve as stepping-stone corridors and transitional regions instead of permanent sites. The timely care of the remaining individuals and the long-term survival of the Bačka region population require efficient management and monitoring of the steppe habitat development phases. Therefore, habitat mapping is one of the main sub-tasks that will enable the successful reintroduction of EGS to future revitalized sites.

### Materials

**Fig 3:**
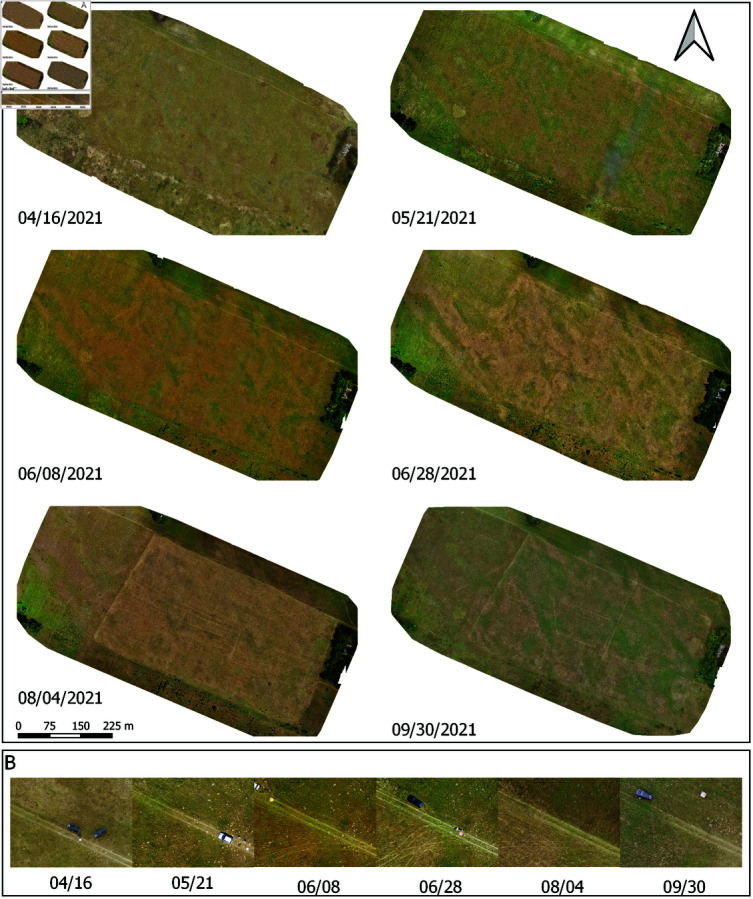
Orthmosaics of the test site illustrating seasonality effect. A: April until September. B: Time lapse of the same spot from which UAV campaigns were initiated.

For this study, we collected two types of data during several fieldwork campaigns organized at the described test site throughout the vegetative season in 2021, Table 1. Research workflow details are given in Fig 2. Collected material regards observations and measurements of the vegetative environment at the given site according to standard phytocoenological practice, S2 Table and the high-resolution UAV images of the same area in the visible light domain, Fig 3.

**Table 1:** Data collection dynamics.

	Fieldwork campaigns (day of month in 2021)						
Type of campaign	April	May	June	July	August	September	in total
vegetation survey	-	14	7; 27	16	4	7; 30	7
UAV acquisition	16	21	8; 28	-	4	30	6

#### UAV image acquisitions

Alongside the recording of phytocoenological releveés, we also performed UAV data acquisitions over the selected test site according to dates reported in Table 1. For imaging was used the first generation of the commercial DJI Inspire UAV, equipped with a Zenmuse X3 RGB camera, an onboard active image stabilization system, and a high-precision GPS receiver with horizontal hovering accuracy of 1 ∕ ( 1 − *ρ* ) = *σ*2.5 m. Data were recorded at spatial resolution of 4000x3000 pixels and the spectral resolution of 3 channels (RGB) per image. Each true color aerial was made with 8-bit/pixel precision and recorded in an uncompressed georeferenced file format. For mission planning we used the “Pix4D capture” application with four connected consecutive rectangular mission grids and acquired around 350 nadir images per one field visit. The mission covered an area of approximately 20 ha with front and side overlap between images of 80%. The flight altitude of 100 m resulted in the GSD of 4 cm per pixel. Orthomosaics of the selected test site were created using “Pix4D mapper” photogrammetry software based on generated digital surface model (DSM) and coregistration using ground control points. Acquisitions were made in sunny weather, mainly at the time close to the local Sun culmination. In total, there were 6 UAV campaigns from April until September, Table 1. Illustrations of generated orthomosaics and seasonality effects are shown in Fig 3, where the image timeline of a small detail illustrates the change of the scene appearance through time.

#### In-situ vegetation survey

Phytocoenological relevée data were recorded inside the selected experimental plots distributed across the site. According to [58], specific phytocoenological data regards the number of species, the percentage cover of dominant species and bare or usurped soil surface. Plant species determination was done manually, on the field, or later, in the lab, according to [59–61] and [62] and reviewed using WFO Plant List (available at: https://wfoplantlist.org/plant-list). Complete Latin names of identified species are provided in the Supporting information: S1 Table, alongside the data collected for each plot during each sampling round, S2 Table. Identification and description of habitat types at the given location was performed according to dominating and characteristic species of the local vegetative environment. This type of habitat-type classification, i.e., classification system based on the description of vegetation types, is also the one suggested and used in the original Habitat Directive [30], as well as in the Natura 2000 network of protected natural areas in the EU [63]. Fieldwork campaigns involving vegetation surveys were organized periodically during 2021, from May until September, according to the schedule described in Table 1. The in-situ measurements and UAV images were taken on the same day or two neighboring dates, depending on weather conditions.

**Fig 4:**
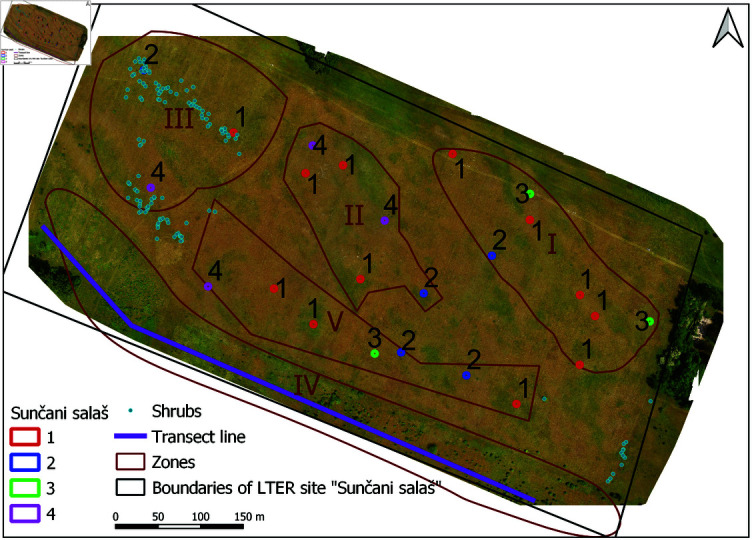
Locations of 1m x 1m vegetation sampling plots and the transect line. According to Table 9 assigned habitat types are: 1–Young steppe I (E1.2C1); 2–Young steppe II (E1.2C2); 3–Bare / fallow land (I1.51); 4–Young steppe I (E1.2C2), while the Forest Steppe I (G1.4) was sampled along the transect line (violet). Small blue dots represent shrubs. Five distinct observation Zones (I–V) are marked in brown.

The collected ground truth data from vegetation survey plots were used to design an automated image classification system that maps corresponding UAV image pixels into one of identified habitat types, i.e. image classification categories. Position of these in-situ research plots and corresponding field observation zones are depicted in Fig 4. Final results of the conducted field research are summarized in Tables 6, 7, 8, and 9.

### Methods

An overview of applied research methodology is depicted in Fig 2. It can be seen that the development of the proposed image classification model was followed by an in-situ vegetation study, which consisted of identifying and characterizing specific vegetation properties that are related to conservation, management and renewal of the EGS population at the given test site. Thus, the specific findings of the in-situ phytocoenological research, presented in the Results section, were an integral part of the designed UAV mapping procedures.

For each labeled pixel in the scene an appropriate description based on UAV image was derived by analyzing RGB values of the surrounding pixels inside the square analysis window around it. Such data were used for supervised learning of pixel-based classifier that was later deployed to whole image (time series). We have considered different spatial and spectral descriptors, their time series, and combinations with varying success. In the following we provide more details regarding conducted image classification experiments – UAV mapping of plant mixtures that were identified as characteristic for certain habitat types, i.e. classification categories.

Regarding the research permits obtained from the local Palić-Ludaš Public enterprise, we would like to point out that all fieldwork was conducted in the agreement and according to valuable suggestions provided by the local ranger service.

#### Classification approach

**Fig 5:**
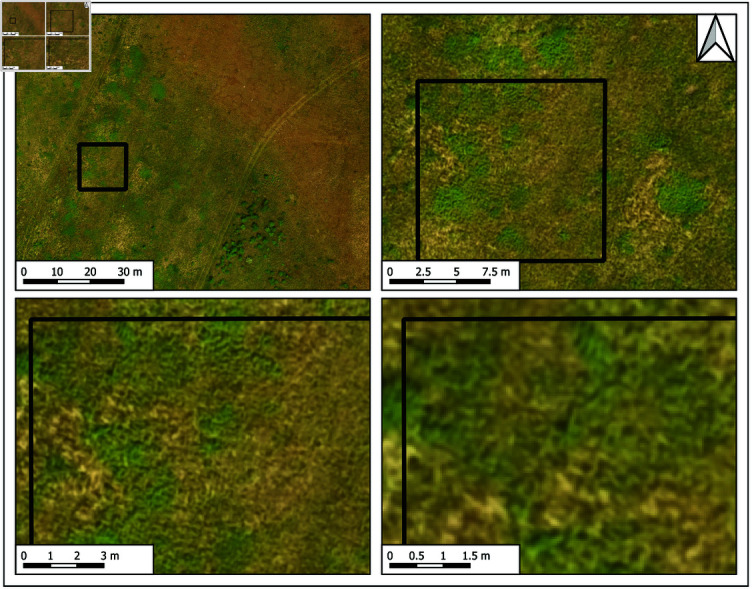
Spatial details in the acquired UAV images. Same part of the scene at different zoom levels (image from the end of June).

The level of spatial details in acquired RGB images is illustrated in Fig 5. It can be seen that despite high GSD resolution of 4 cm per pixel (25x25 pixels inside 1m x 1m vegetation sampling plot), individual plants in the mixture were not visually distinguishable in the image. Since the total number of research plots was relatively small (24 plots shown in Fig 4, i.e. defined in S1 Fig and S2 Table), number of labeled samples per each UAV image was also relatively low. It should be mentioned that each of the plots was continuously monitored by periodic in-situ vegetation surveys during the whole season in order determine the habitat type label of each plot. Thus, due to relatively small number and extent of labeled image patches it was not possible to apply an object-based image analysis (OBIA), and instead a pixel-based classification was performed. Such design choice was mostly influenced by the fact that vegetation surveys and parameters of UAV acquittions were not jointly planned from the beginning of research study. On the other hand, design of mapping system under described unfavourable conditions of insufficient level of details, spectral resolution and number of ground-truth samples, resembles many use case scenarios in which ranger services have limited time and material resources, e.g. scenarios preferring UAV acquisitions with higher flight altitudes in order to achieve larger spatial coverage. Therefore, instead of an end-to-end machine learning, a descriptor based approach for classification of plant mixtures around each sampling point (image pixel) was adopted.

#### Classification categories

Mapped habitat types included:

- Class *C_0_* or “Steppe”, encompassing Young steppes I and Young steppe II types;
- Class *C_1_* or “Shrubs”;
- Class *C_2_* or “Forest–Steppe”, consisting of Forest–Steppe I; and
- Class *C_3_* or “Bare/fallow land”, encompassing Fallow land.

As can be seen, listed categories *C_0_*-*C_3_* that were the subject of image classification represent aggregated or redefined versions of the original habitat types that were defined and identified as the result of extensive fieldwork at the test site (for more details please see the Habitat development in the Results section). Thus, due to lack of fine spatial details that would allow for visual identification of individual plant parts, and a relatively small number of sampling plots per each habitat type, we redefined original recognition problem by combining some of the identified habitat types from Table 9 into one of four image classification categories *C_0_*-*C_3_*.

#### Image sampling

**Fig 6:**
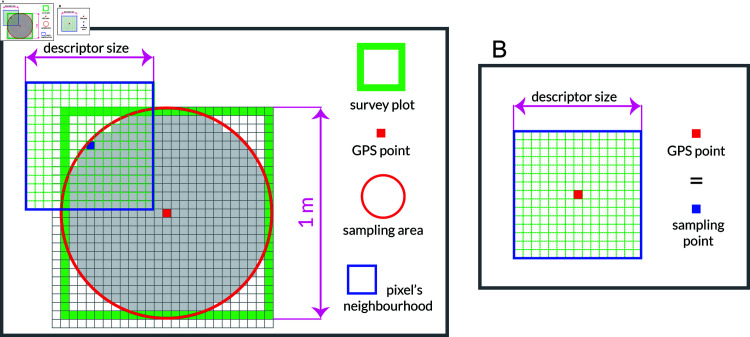
Sampling of the ground-truth image data. A: For class *C_0_* or “Steppe”, each pixel inside the inscribed circle was used as a source of labeled data. B: In the case of *C_1_*, *C_2_*, and *C_3_*, only the point sampling of the central GPS position was performed.

Image sampling procedures during feature extraction were generally adapted to specific requirements of certain image descriptor. In the case of ground-truth (GT) or labeled samples that were used for model training and validation, there were also additional adaptations that were reflecting the specificities of the habitat type survey, i.e. differences in the way how vegetation surveys were conducted over 24 research plots designated by number symbols in Fig 4, on one side, and the GT samples taken along the transect line or over the areas dominated by shrubs in Fig 4, on the other. GT sampling of UAV images was based on precise GPS locations of research plots listed in S2 Table, i.e. Table 9, and points corresponding to the transect line and shrubs in Fig 4. This is illustrated in Fig 6, where two different adaptations of GT sampling are shown.

Besides the relative position of GPS measurement and GT sampling point, Fig 6 also illustrates the spatial analysis window that defines the spatial support of the corresponding pixel descriptor. Since the exact orientation of 1m x 1m research plots was not recorded (non-oriented plots), sampling of individual pixels belonging to class *C_0_* “Steppe” was performed as illustrated in Fig 6 A, by sampling the area corresponding to an inscribed circle with radius equal to 1 m. Depending on feature extraction method, descriptor size was predefined or optimized based on the results of conducted classification experiments. On the other hand, for the categories *C_1_*, *C_2_*, and *C_3_*, GT sampling was performed by extraction of one labeled pixel and its neighbourhood – corresponding to the recorded GPS coordinate of the individual point or plot’s center, Fig 6 B. It comes from the way how instances of “Shrubs”, “Forest–Steppe” and “Bare/fallow land” were sampled in the field, and heterogeneity of plots in the case of the “Bare/fallow land”.

“Shrubs” were not dominant in any of the analyzed vegetation survey plots, but were significantly present in several places across the test site, Table 9. Therefore, GT data for class *C_1_* were taken based on the in-situ located GPS points, indicated by blue dots in Fig 4. Similarly, point sampling of images was also performed for classes *C_2_* and *C_3_*. The reason was that the sampling plots corresponding to *C_3_* “Bare/fallow land” had significantly heterogeneous structure, which prevented finer pixel sampling within the 1m x 1m area (neighbourhood of the plot center was mostly non-uniform). In order to increase the number of point samples for the class *C_3_* (originally only three plots), an additional sampling of bare land based on the photointerpretation of the UAV images was performed. This provided 30 samples and was possible because the “Bare/fallow land” class was easily distinguishable in UAV images and did not require detailed vegetation survey on the ground. GT for the class *C_2_* “Forest–Steppe” was taken by equidistant sampling along the transect line, which resulted in 134 sampling points, Fig 4.

Taking into account that GSD was 4 cm, described sampling methodology led to significant number of labeled feature vectors for class *C_0_* based on relatively small number of research plots, but also brought additional difficulties in imbalanced sample size over GT categories. Nevertheless, described sampling methodology proved to be effective for the design of the proposed descriptor based image classifier. The same feature extraction process was repeated for each image in the UAV time series.

#### Model validation

When it comes to validation of the proposed mapping procedures, it was performed both by objective, quantitative criteria, and by visual, qualitative assessment of produced habitat type maps, followed by comparisons with collected in-situ data. Classification performance of different mapping models (Fig 2) was measured by the means of statistical cross-validation, in a manner that resembles the standard leave-one-out approach [64].

Performance estimates of each analyzed classification model were thus obtained as the averages of several individual classification experiments in which one of the samples is held out of the sample during the model training, and later on used only for testing. However, since the GT samples or feature vectors coming from the same 1m x 1m plot of class *C_0_* were expected to have low intra-class variance, instead of usual leave-one-out approach a slight modification was applied. Namely, all GT samples from the same *C_0_* sampling plot were always used together in the individual leave-one-out experiments (as part of the same training or test set subset). Thus, for each test involving *C_0_* samples, all GT samples from the same *C_0_* plot were always used together for testing, and the rest of *C_0_* plots (their GT samples) were used for training. Similarly, in order to perform the same type of leave-one-out cross-validation for classes *C_1_*, *C_2_*, and *C_3_*, instead of shuffling individual GT samples, each set of the class specific GT samples was first randomly partitioned into five disjoint subsets, and only after that these subsets were moved between cross-validation training and test sets in each leave-one-out experiment.

The results of experimental evaluations were reported in a standard way [65] by quantitative measures of overall accuracy, Cohen’s kappa coefficient [66], individual user’s accuracy (precision), and producer’s accuracy or recall [67], per each of defined pixel categories. Outcomes of different experiments were also tested against each other for the presence of statistically significant difference.

Habitat related results that were used for defining image classification problem and GT sampling were validated by the means of standard phytocoenological practice and based on continuously collected in-situ data during the whole growing season (S2 Table).

#### Initial set of experiments

As the first attempt to capture spatial context of each pixel, a set of classification experiments involving densely computed local image descriptors of textures [68] and edges was performed. Although these experiments required substantial computation, constructed feature vectors based on local binary patterns (LBPs) [69,70], histograms of oriented gradients (HOGs) [71,72], and Fisher vectors (FVs) [73,74] of directed Gabor filter repsonses [75,76], did not provide enough discriminatory information in order to achieve an acceptable classification performance. Nevertheless, for the sake of completeness we have decided to briefly report considered descriptors and point out some of observed peculiarities. Number of features per pixel (FPP) was varying depending on dimensionality of each descriptor, however the general finding was that due to the high spatial resolution of UAV images, precomputation of the corresponding feature maps is not recommended and on-the-fly processing should be performed.

Edge descriptors in the form of HOGs were computed over the spatial windows of 32x32 pixels (1.28m x 1.28m), with cell size of 8x8 pixels and $L_{2}$ normalization blocks of 2x2 cells. By discretization of edge orientations into 9 uniform bins, described procedure resulted in 324 FPP. Over the same window, histograms of uniform LBPs at three different scales, for radius *ρσ* = 1 − *σ* in total gave 177 textural FPP. However, after being applied to analyzed pixel-based classification problem, described descriptors did not perform well and were abandoned. As an alternative multiscale texture characterization, the set of directed Gabor filters at 8 orientations and 5 scales, providing 40 FPP was also considered, but resulted in low classification accuracy. Since UAV images were acquired at a relatively high spatial resolution, it was expected that discriminative information was contained in the grassland texture. Therefore, in order to perform an unsupervised learning of optimal texture representation, in a style similar to [76] and [77], the idea was to utilize an advanced Fisher encoding scheme and thus aggregate Gabor filter responses over the analysis window of size 128x128 (5m x 5m) around each pixel. Since the modeling of multivariate distribution of original 128x128 40 dimensional feature vectors consisted of fitting a Gaussian mixture model with K=10 Gaussians and diagonal covariance matrices, the overall dimensionality of generated Fisher vectors resulted in 800 FPP. This was much smaller in comparison to the initial 128x128x40 Gabor filter responses (i.e. feature maps) assigned to each pixel. However, after initial implementation and testing, it came out that described Fisher vector approach would not be computationally feasible in practice due to large size of UAV orthomosaic, and especially taking into account UAV time series. Therefore, FVs method was also abandoned.

#### Proposed high resolution descriptors

Finally, we were able to successfully solve the remote sensing (RS) classification problem by utilizing spatial descriptors in the form of gray-level co-occurrence matrices (GLCMs), which were computed from the grayscales of UAV RGB images for the square spatial neighbourhood around each pixel. These types of features have been utilized in the remote sensing classification tasks since the very beginnings [78,79], and were also successfully applied in the more recent grassland classification studies like [39,40]. These texture descriptors were computed in a multiscale manner (for different filter sizes), and different types of texture statistics. For processing was utilized an efficient implementation provided in [80].

GLCM texture features listed in Table 2 correspond to the expected values of different quantities inside the spatial analysis window. These are computed by averaging over the pixel pairs distribution, i.e. by GLCMs and their marginals. Considered quantities include: energy or texture uniformity, entropy, correlation, homogeneity, contrast, cluster shade, cluster prominence, Haralick correlation, mean, variance, dissimilarity, sum average, sum variance, sum entropy, diference of entropies, difference of variances, and two information measures of correlation. GLCM features of each pixels were computed for different filter sizes of: 17x17; 31x31; and 61x61 pixels, which correspond to the spatial extent of 0.7 m x 0.7 m; 1.2 m x 1.2 m; and 2.4 m x 2.4 m around each pixel, respectively. Also, the pixel pair distributions over which expectations of listed quantities were computed were determined by the pairs of pixels at the $L^{\infty }$ distance of: 4; 8; and 16 pixels, respectively, positioned at the 45° relative to each other. More information about each of listed quantities are available in [80].

**Fig 7:**
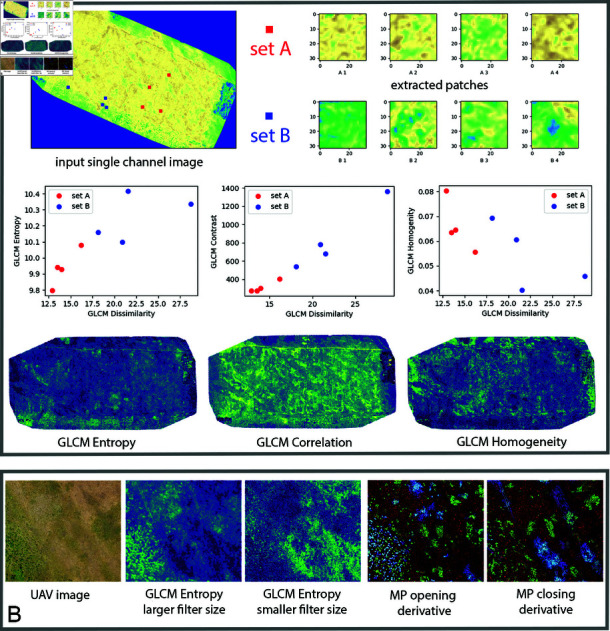
Proposed texture and morphological image descriptors. A: Comparison of three GLCM texture features: entropy, correlation, and homegeneity. Scatter plots indicate good discriminability between spatial neihbourhoods of the pixels corresponding to different types of vegetation patterns (patches denoted as sets *A* and *B* in the upper-right part of the figure). B: Morphological profile (MP) features, corresponding to MP derivatives of the morphological opening and closing by reconstruction, as compared to the GLCM entropy feature maps of the same image area (GLCM entropy variants corresponding to different filter sizes).

Some of the proposed GLCM features are illustrated in Fig 7A, where the examples of the corresponding feature maps of GLCM entropy, GLCM correlation, and GLCM homogeneity are visually compared. It also indicates their discriminative capabilities, by comparing GLCM representations of 8 random patches corresponding to two different types of vegetation patterns (denoted as set A, and set B).

**Table 2:** Proposed GLCM texture descriptors. Computed for 3 different scales (analysis window sizes) by averaging over the pixel pairs at distance of $L^{\infty }$ and positioned at 45° relative to each other. In total 54 FPP.

Feature type	Abbreviation	size #1	size #2	size #3
energy or uniformity	GLCM energy			
entropy	GLCM entropy			
correlation	GLCM correlation			
homogeneity	GLCM homogeneity			
contrast	GLCM contrast			
cluster shade	GLCM clShade			
cluster prominence	GLCM clProminence			
Haralick correlation	GLCM Hcorrelation	17x17	31x31	61x61
mean	GLCM mean	pixels	pixels	pixels
variance	GLCM variance			
dissimilarity	GLCM diss	0.7x0.7	1.2x1.2	2.4x2.4
sum average	GLCM sumAvg	m	m	m
sum variance	GLCM sumVar			
sum entropy	GLCM sumEnt	$L^{\infty }\;=\;4$	$L^{\infty }\;=\;8$	$L^{\infty }\;=\;16$
diff. of entropies	GLCM diffOfEntropies			
diff. of variances	GLCM diffOfVar			
inf. measure of corr.#1	GLCM infMcorr1			
inf. measure of corr. #2	GLCM infMcorr2			

In total, there were D=54 GLCM *texture* features per pixel (FPP), corresponding to 18 different quantities computed over 3 different scales (filter sizes) for each UAV image in the times series.

Besides GLCMs, we also utilized morphological profile (MP) features [81], and their efficient implementation in [80]. First, MPs of opening and closing by reconstruction at 5 scales (filter sizes) were generated for each UAV image. Then, MP derivatives were computed for each of these 5 image stacks (5 MP obtained by opening provide 4 MP opening derivatives, similarly also for closing). Thus, 8 feature maps of MP derivatives were obtained for each UAV image (we note that plain opening and closing MPs were not used, only their derivatives). In addition, one “characteristic” of computed MP derivatives was extracted (one for opening, and one for closing). This produced 2 additional feature maps, which resulted in 10 unique values per each pixel. At the end, one more value of MP classification was added per each pixel, giving the final MP feature vector dimensionality of D=11 *morphological* FPP, Table 3. Type of information provided by the proposed set of MP features is illustrated in Fig 7B, where the MP derivatives of opening and closing for the three smallest filter sizes are visualized (shown as the corresponding pseudocolor composites consisting of the computed feature values).

**Table 3:** Proposed morphological descriptors. Morphological opening and closing by reconstruction at *σ* = 1 different scales (filter sizes) with ball shaped filter of radius  ∼  pixels and step size of ${\text{r}}_{\text{s}}=1$, $\left(\text{r},\text{s},{\text{r}}_{\text{s}})=(5,5,1\right)$. In total 11 FPP.

Feature type	Abbreviation
MP derivatives (opening), ≈	MPDerivOpening(5,5,1)scale *N*
MP derivatives (closing), ≈	MPDerivClosing(5,5,1)scale *N*
MP characteristic (opening)	MPCharacteristicOpening(5,5,1)
MP characteristic (closing)	MPCharacteristicClosing(5,5,1)
MP classification	MPClassification(5,5,1)

^a^*N* refers to scale of derivatives (plain MPs were used only for derivatives computation).

Besides original RGB values, additional 6 features reported in Table 4 were also utilized in classification experiments, resulting in 9 *spectral* features per pixel (FPP).

**Table 4:** Proposed spectral descriptors. Computed from 3-channel RGB images according to the provided references. In total 9 FPP.

Feature type	Abbreviation	Reference
RGB values	R,G,B	-
soil coloration index	colRG	[82]
BG coloration	colBG	-
green-red ratio	GRratio	[83]
blue-green ratio	BGratio	-
pixel brightness	pGluminance	[83]
green leaf index	GreenLeaf	[84]

Most of classification experiments were performed using Xeon E5-2690V4 machine with 28 cores and 380 GB of memory, without any specific performance optimizations except customized parallelization of different processing tasks. Parallel execution and batch processing was necessary most of the time due to a relatively large size of UAV orthomosaics (approximately 20500x13800 pixels) and the presence of their time series.

As a classifier with adaptive learning capacity and robust to overfitting, random forest model (RF) [85] based on ensemble of decision trees [86,87] was used in all experiments.

In order to improve classification and ease model deployment, an additional feature selection procedure based on [88] was performed. By selection of an optimal subset of relevant features from the described image descriptors, it substantially reduced the computational requirements of the proposed UAV mapping, and also led to better model performance. The VSURF two-step feature selection procedure [89] consists of the analysis of the performance statistics of ensembles of RF classification models. First, the importance ranking and elimination of redundant features is done based on nonparametric statistics produced by generated RF instances. Then, in the second step, the remaining features that could be relevant for classification problem are further analyzed and ranked based on their interpretation value. Finally, the subset of features contributing mostly to the prediction objective was adopted as the final feature set.

Introduction of the described feature selection was mostly motivated by the fact that model performance was highly influenced by the temporal dynamics of grassland vegetation. Thus, in all experiments models based on the entire UAV time series exhibited significantly better results. A disadvantage was that the applied fusion of temporal information was done at the feature level, by concatenation of descriptors from individual images in UAV time series, leading to high dimensionality of feature vectors.

In all experiments, UAV image time series were classified based on adopted list of features and RF models of the same type (number of decision trees in the ensemble, tree depth, feature sampling and splitting criteria in tree nodes). Such models were compared against each other based on different sets of input features (textural, morphological, spectral, and their combinations) and performance measures (with and without feature selection). Thus, depending on the type of conducted experiments, dimensionality *D* of the features was varying. At the end, the best performing model was selected.

## Results

Obtained results encompass two tracks of the conducted research, Fig 2. The first one related to EGS habitat findings, based on extensive in-situ vegetation analyses, and the second one related to the proposed high resolution image descriptors for UAV mapping.

Although these two tracks were conducted in parallel, initial decisions concerning the type of UAV imaging (like the flight height and mission duration), as well as vegetation monitoring (like the number of research plots, and the time and number of field visits and UAV acquisitions) was not possible to make based on previous experience, which had profound impact on the characteristics of the defined mapping problem. On the other hand, from the beginning, development of UAV mapping solution was motivated by the hypothesis that it would be possible to perform mapping of complex plant mixtures representing different habitat types based only on high resolution RGB images, without the need for deployment of more sophisticated multispectral sensors. The hypothesis was also reflecting the assumption that the potential users, like the local ranger service, will not have multispectral instruments. Therefore, spatial and temporal domains of UAV images were considered as the main sources of possible information for successful mapping model. Since one of the requirements was also to optimize for the flight time, level of spatial details was determined by the combination of camera’s spatial resolution and the flight altitude. The consequence was that besides the relatively small number of GT samples, classification problem was also made difficult by the lack of more fine-scale spatial information about individual plants. In the following we present original research results of both study tracks, and provide more details regarding their interplay.

### Habitat characterization

Habitat characterization consisted of identification of observation zones and the key habitat types (biotopes). Based on spatial variations in habitat heterogeneity, observed visually from the first UAV imaging campaign in April 2021, Table 1, in total there were identified and recognized 5 distinct observation zones at the study site, which differ in the area they occupy, as reported in Table 5.

**Table 5:** Identified observation zones and their characteristics.

	Area (ha)	Number of survey plots	Number of reference points
Zone 1	2.8	6	300
Zone 2	2.1	6	300
Zone 3	3.5	3	150
Zone 4	5.5	transect	200
Zone 5	2.6	9	450

Within four of these zones (1-3; 5) which correspond to the open grassland area, we set up a different number of 1x1 m research plots (24 in total) in proportion to the heterogeneity of the surface. The plots were set up at mutual distances of at least 5 m one from each other, and positioned by the GNSS Leica GS07 GPS rover with real-time kinematic (RTK) support and CS20 controller, providing horizontal positioning precision of the center point at 2.5 cm. Inside each of these non-oriented 1x1 m plots phytocoenological releveés were recorded. In addition, we defined a buffer area with a 5 m radius around each studied plot. Inside this area, we marked reference points designating dominant species using the same GPS antenna, in order to provide additional data for future reference. In the Forest–Steppe zone, designated as Zone 4 in Table 5, instead of plot surveys, a linear transect method was applied to determine the vegetation composition. The transect was done because zone 4, although a part of the study site, was not a target habitat for us, i.e., not an open grassland. Furthermore, zone 4 occupies a linear shape, which makes the transect method more appropriate to use.

Based on fieldwork vegetation data and, according to [90], we classified Sunčani salaš vegetation into appropriate EUNIS classes [91] and assessed its conservation status. These classes were then translated into site-specific key habitat types for site management purposes. The reasoning behind such decision was also that the types were easier to designate and monitor on the ground from the perspective of site managers, results in Table 6). It was done by following the methodology presented in [3].

**Table 6:** Site specific steppe habitat types and their quality in relation to the EGS.

Habitat type	The definition used to describe the steppe habitat type
Steppe	Vegetation that grows on a sandy substrate, dry throughout most of the year. *Festuca rupicola* is the dominant plant and can close up to 100 % assembly on a radius greater than 50 cm surface. For this reason, vegetation transition to wooden-shrub form on the western part of the habitat can be noticed, Fig 3B.
Young steppe	Vegetation is characterized by the presence of *F. rupicola* > 6 [58] (50 %) at a 50 cm radius.
Non-managed young steppe	Vegetation is characterized by the presence of shrubs: *Rosa canina*, *Crataegus monogyna*, and *Rubus sp.* due to a lack of maintenance of the open habitat.
Forest-steppe	Vegetation is characterized by wooden and rye forms with species: *Populus alba*, *Salix sp*, and *Fraxinus ornus*
**Habitat suitability**	**Definitions used to evaluate quality in relation to the EGS**
Vegetation optimal for the EGS	Habitat optimal for EGS should meet the following criteria: a) Presence of > 20 % *Achillea millefolium* or Fabaceae (*Medicago sp.*, *Trifolium sp.*, *Vicia sp.*, etc) b) either Poaceae coverage is 10-60 % or *Poa sp.* and *Festuca sp.* are present, the latter with at least 50 % cover c) *Cirsium sp.*, *Carduus sp.*, *Rosa canina*, or other tall species account for less than 20 % cover d) *Potentilla argentea*, *P. incana*, *Plantago sp.*, *Veronica sp.*, and *Asteraceae* are present.
Vegetation suboptimal for the EGS	Areas with species unfavorable for the EGS (covering, usually, bare ground and (mechanically) usurped surfaces; invasive species and shrubs): *Ambrosia artemisifolia*, *Amaranthus retroflexus*, *Setaria viridis*, *Cynodon dactylon*, *Asclepias syriaca*, *Erigeron canadensis*, *Carduus nutans*, *Bromus sterilis*

### Habitat development

Identification of habitat development phases (or stages) was performed by combining in-situ vegetation survey and information on the natural vegetation of the area (sans habitat destruction in the past) available from the literature [59,90]. As one of the original research results we have proposed distinct habitat development phases, as described in Table 7. Each one is defined using the set of indicator species, which reflect the site-specific expert opinion [3].

**Table 7:** Identified steppe development phases on the grassland surface of Sunčani salaš.

	Site-specific definitions of the habitat development phases (stages) ^a^
Fallow land (stage I)	Characterized by invasive species and weeds, for example: *Ambrosia artemisiifolia*, *Amaranthus retroflexus*, *Setaria viridis*, *Cynodon dactylon*, *Asclepias syriaca*, *Erigeron canadensis*, as well as *Carduus nutans* and *Bromus sterilis* These species also occur in areas usurped by wild boars and where the cover is minor (e.g., open sand).
Young steppe I (stage II)	Characterized by the presence of *Festuca rupicola* with a minimum cover of 50 %, *Cynodon dactylon* occurs with greater representation than in the previous phase (20-30 %); the presence of *Trifolium arvense*, *Medicago minima*, *Vicia hirsuta*; *Asperula sp* and *Verbascum sp* but also *Achillea millefolium* and *Dianthus giganteiformis* subsp. *pontederae* present with a maximal cover of up to 10%.
Young steppe II (stage III)	Characterized by the presence of *Festuca rupicola* with cover up to 90% (it has almost completely closed the habitat), and the presence of *Bothriochloa ischaemum* may be detected, there is also *Calamagrostis epigejos* and *Stipa pennata* on the edges of the grassy area that is in the vicinity of the forest-steppe zone. In the lower percentage, there is also *Verbascum sp.*
Steppe (stage IV)	The most distinctive is the cover or complete closure of habitats with *Festuca rupicola*, *Galium glaucum*, *Stipa pennata*, *S. capillata* and *Dianthus giganteiformis subsp. pontederae*. In addition, the *Rhinanto-Festucetum* association is expected to be detected on the sand steppe, with a fully closed canopy consisting of *Rhinanthus borbasii* and *Festuca pratensis*. This association was present in the habitat before the plowing. The emergence of species *Poa prantensis*, *Achillea milefollium*, *Dianthus giganteiformis subsp. pontederae*, *Dactylis glomerata* is expected in this habitat as well as in the steppe on the loess, but the presence of *Verbascum phoeniceum*, *Ononis spinosa*, *Elymus repens*, *Allium scorodoprasum* as well as *Plantago media* further confirms that Sunčani salaš is a sandy steppe habitat.
Forest-steppe I	Characterized by 50 % growth of, mostly, *Crataegus monogyna*, and other woody plants and grassy formations.
Forest-steppe II	Should be characterized by *Populus alba*, *Salix sp.* and *Fraxinus ornus*, but invasive species: *Robinia pseudoacacia*, *Celtis occidentalis* and *Gleditsia triacanthos* are present in the first phase of forest-steppe (as young plants). Those three invasive species came after the man-made forest plantations in the Subotica sands area were completed. These are expected to spread due to climate change and suitable environmental conditions in the area. The second phase is characterized by 70 % to 100 % of *Crataegus monogyna*. After *Crataegus monogyna*, 3 invasive species given above along with others take over the cover and lock the habitat into a forest assembly.
Non-managed young steppe and shrubs	If the open habitat is not maintained (by mowing and grazing), open grassland will go into wooden-shrub form regardless of the development phase. In this case, the vegetation is characteristically like the greenery of macchia and gariga in the Mediterranean. This vegetation is characterized by shrubs and spiky vegetation, and in the temperate environment, it most likely occurs due to climate change and soil nitrification. The following species occur: *Rosa canina*, *Crataegus monogyna*, and *Rubus sp.*

^a^A habitat is considered a steppe if at least 20 % of vegetation is in succession stages II, III or IV and no more than 30 % is covered in ruderal vegetation or invasive species. If any of the criteria is not met, the habitat is no longer a steppe.

As indicators of the habitat development process, we propose tracking the following quantities and quality indicators for each habitat development phase for EGS, Table 8:

- Extent (total surface) of the mapped habitat succession phase (stage);
- Suitability of the development phase for supporting the EGS individuals i.e., provide habitat, resources;
- Degree of regeneration, compared to the initial state (arable land).

**Table 8:** Proposed indicators for monitoring steppe development phases.

State indicators	Steppe grassland habitat at Sunčani salaš	
Extent of steppe development phase	Baseline state	The extent of the development phase of the steppe that is identified in the habitat
Quality of steppe development phase	Lower limit definition	> 20 % of the habitat is under the steppe vegetation in succession stages II, III, or IV
	Upper limit definition	< 30 % of the habitat is under ruderal vegetation or invasive species in stage I

By monitoring these characteristics, we can assess the direction of habitat restoration. Regarding the extent of vegetation surface corresponding to each identified revitalization phase, Table 7, we have proposed an approach based on the spatial distribution map derived from the UAV images acquired during the vegetative season. Thus, by utilizing the proposed image classification algorithm based on RGB image data, the extent of manually identified habitat development phases was quantified in an automated manner.

The suitability or quality indicator for the state of each steppe habitat type in relation to the EGS population was determined based on the composition of plant species. It was done by extensive literature review and expert opinion concerning the primary conservation goal at the site: translocation and maintenance of a viable EGS population and improved site management. However, before establishing the suitability of the steppe habitat types concerning the EGS, the assessment of whether the habitat type is steppe or not was required based on the two necessary quality criteria described in the footnote of Table 7.

### EGS habitat renewal

Results describing EGS habitat renewal process at the Sunčani salaš study site were as follows: out of the four proposed succession stages of the open grassland from an arable land into a steppe habitat, three were confirmed during the fieldwork campaigns - Fallow land (stage I), Young steppe I (stage II), and Young steppe II (stage III); with one part of the site characterized as not maintained and with a substantial presence of shrubs.

The most significant part of the open grassland habitat at the site was in the second succession stage: Young steppe I, with *Festuca rupicola*, characteristic for steppe habitats on the sand, dominating the area almost entirely. As a result of the influx from the neighboring Forest–Steppe zone, *Stipa pennata* was present in the southwestern part of the site, so we consider this part of the site to be in the III succession phase (Young steppe II). Due to lack of management, the western part was characterized by the presence of shrubs (*Crataegus monogyna*, *Rubus sp*, *Rosa canina*). Finally, the southern part of the site represents the first phase of the succession to Forest–Steppe habitat (Forest–Steppe I). Here, approximately 50% of the habitat is under *Crataegus monogyna*. The three plots characterized as fallow land were located on the edges of the open grassland (points 6, 19, 24 in the S1 Fig). Similarly, edge plots 3, 18, and 20 (S1 Fig) were at some point throughout the sampling period characterized by fallow land vegetation. The species with the highest coverage within the plots classified as Fallow land (stage I) is the invasive *Asclepias syriaca*, S2 Table in the Supporting information.

Based on the presence of characteristic species, we identified four main habitat types according to EUNIS (European nature information system) classification: Pannonic loess steppe grassland (E1.2C1 and E1.2C2), Pannonic sandy steppes (E1.2F4), Broadleaved deciduous woodland (G1.4) and Bare tilled land (I1.51). Of all detected habitat types, Pannonic loess steppe grassland (E1.2C1 and E1.2C2) and Pannonic sandy steppes (E1.2F4) are of conservation importance and are listed in the Annex I of the Habitats Directive [30]. Details on the key species in these habitat types and how these were translated into habitat development phases at the site are given in Table 9.

**Table 9:** Overview of the main habitat types and vegetation characteristics at the site.

Habitat type (EUNIS level IV)	Key atributes	Research plots	Local-specific definition of succession phase	Key atributes	Total research plots
E1.2C - Pannonic loess steppic grassland					
E1.2C1 - Pannonic loess steppes^a^	*Festuca rupicola* dominates	12	Young steppe I	*Festuca rupicola*, *Verbascum phoeniceum*, *Euphorbia cyparissias*	16
E1.2C2 - Pannonic tall forb meadow-steppes	*Bothriochloa ischaemum* dominates	4	Young steppe I	*Bothriochloa ischaemum, Potentilla incana, Potentilla argentea*	
E1.2F - Pannonic sandy steppes					
E1.2F4 - Pannonic closed sand steppes	*Festuca rupicola* dominates	5	Young steppe II	*Stipa capillata, Stipa pennata, Festuca rupicola, Poa bulbosa*	5
G1- Broadleaved deciduous woodland					
G1.4 - Broadleaf swamp forest on non-acid peat	*Fraxinus ornus* and *Ulmus campestris* dominate	^ b^	Forrest-steppe I	*Ulmus campestris, Fraxinus ornus, Crataegus monogyna, Stipa pennata*	^b^
I- Regularly or recently cultivated agricultural, horticultural and domestic habitats					
I1.51 - Bare tilled land	20-50 % bare ground	3	Fallow land	*Cynodon dactylon Ambrosia artemisifolia, Asclepias syriaca*	3
Other					
Shrubs	*Crataegus monogyna* and *Rosa canina* dominate	3^ c^	Western part of the site overgrowing due to lack of management	*Rosa canina, Rubus caesius, Rubus praecox, Crataegus monogyna*	3

^a^The presence of loess in the sand is detected by carbonate concentrations. Two habitat types share a number of characteristic species. ^b^Transect method was applied instead of plots. ^c^Shrubs do not dominate any specific plots, but are significantly present within three plots in the western part of the site.

According to detected species and the quality criteria shown in Table 8, open grassland area of Sunčani salaš characterized as Young steppe (I and II), can be regarded as suitable for the ecological and management needs of the European ground squirrel. Forest–Steppe areas with a significant presence of invasive species, and the non-managed part of the open grassland, on the other hand, do not.

### Habitat type mapping - Image classification models

Descriptor design and motivation for the proposed image classification approach based on UAV image time series have been described in detail in the Methods section. Proposed high resolution descriptors are focusing on spatial information and their temporal fusion in order to overcome low spectral resolution of utilized RGB camera sensor. Experimental results have confirmed initial hypothesis that in the given case (under described UAV imaging and application related conditions) design of image classification model based on RGB measurements is possible, however only in the case when time series information is utilized. Moreover, the results of conducted feature selection experiments have shown that the limited presence of spectral information in RGB image time series still has a significant impact on capturing temporal dynamics of grassland vegetation, especially at times when it manifests in significant colour change. Besides texture, morphological and spectral features, in Methods section were also proposed the corresponding sampling and image classification procedures, which were adapted to the specific requirements of the use case (results of the vegetation study) and the image classification task (classification categories and their visual characteristics).

Results of image classification experiments comparing different types image descriptors (spatial and spectral based), are summarized in Table 10. Model performance was evaluated according to Model validation procedure and measures described in Methods section, which were applied to obtain quantitative and qualitative assessment of mapping results.

**Table 10:** Results of classification experiments for different high resolution descriptors. ^a^ ${\text{C}}_{0}$–“Steppe”, ${\text{C}}_{1}$–“Shrubs”, ${\text{C}}_{2}$–“Forest-steppe”, ${\text{C}}_{3}$–“Bare/fallow land”.

*spectral* descriptors										
	${Ĉ}_{0}$	${Ĉ}_{1}$	${Ĉ}_{2}$	${Ĉ}_{3}$	FN	PA	UA	ACC	*F*	total
*C_0_*	**8319**	82	51	4	137	0.98	0.99	0.97	0.99	
*C_1_*	11	**100**	12	0	23	0.81	0.51	0.99	0.63	Acc=96.97
*C_2_*	64	13	**57**	0	77	0.43	0.47	0.98	0.45	κ=0.55
*C_3_*	26	0	2	**2**	28	0.07	0.33	1.00	0.11	$\kappa_{95}\in \kappa \pm 0.046$
FP	101	95	65	4	avg:	0.57	0.58	0.98	0.54	*D* = 6 ⋅ 9 FPP
**GLCM *texture* descriptors**										
	${Ĉ}_{0}$	${Ĉ}_{1}$	${Ĉ}_{2}$	${Ĉ}_{3}$	FN	PA	UA	ACC	*F*	total
*C_0_*	**7953**	54	449	0	503	0.94	1.00	0.94	0.97	
*C_1_*	12	**93**	17	1	30	0.76	0.59	0.99	0.66	Acc=93.58
*C_2_*	1	10	**118**	5	16	0.88	0.20	0.94	0.33	κ=0.45
*C_3_*	7	0	5	**18**	12	0.60	0.75	1.00	0.67	$\kappa_{95}\in \kappa \pm 0.037$
FP	20	64	471	6	avg:	0.79	0.64	0.97	0.66	D=6⋅54 FPP
***morphological profile* descriptors**										
	${Ĉ}_{0}$	${Ĉ}_{1}$	${Ĉ}_{2}$	${Ĉ}_{3}$	FN	PA	UA	ACC	*F*	total
*C_0_*	**8243**	78	135	0	213	0.97	0.98	0.96	0.98	
*C_1_*	23	**100**	0	0	23	0.81	0.53	0.99	0.64	Acc=95.47
*C_2_*	121	9	**3**	1	131	0.02	0.02	0.97	0.02	κ=0.34
*C_3_*	28	1	0	**1**	29	0.03	0.50	1.00	0.06	$\kappa_{95}\in \kappa \pm 0.048$
FP	172	88	135	1	avg:	0.46	0.51	0.98	0.43	D=6⋅11 FPP
***combined* descriptors (*spectral*+*texture*+*morphological*)**										
	${Ĉ}_{0}$	${Ĉ}_{1}$	${Ĉ}_{2}$	${Ĉ}_{3}$	FN	PA	UA	ACC	*F*	total
*C_0_*	**8410**	8	38	0	46	0.99	1.00	0.99	1.00	
*C_1_*	8	**107**	7	1	16	0.87	0.90	1.00	0.88	Acc=99.06
*C_2_*	2	4	**124**	4	10	0.93	0.72	0.99	0.81	κ=0.86
*C_3_*	7	0	3	**20**	10	0.67	0.80	1.00	0.73	$\kappa_{95}\in \kappa \pm 0.029$
FP	17	12	48	5	avg:	0.86	0.85	1.00	0.85	D=6⋅74 FPP
***combined* descriptors after feature selection**										
	${Ĉ}_{0}$	${Ĉ}_{1}$	${Ĉ}_{2}$	${Ĉ}_{3}$	FN	PA	UA	ACC	*F*	total
*C_0_*	**8216**	160	0	80	240	0.97	1.00	0.97	0.98	
*C_1_*	5	**108**	9	1	15	0.88	0.39	0.98	0.54	Acc=96.83
*C_2_*	0	8	**125**	1	9	0.93	0.92	1	0.93	κ=0.64
*C_3_*	10	1	2	**17**	13	0.57	0.17	0.99	0.26	$\kappa_{95}\in \kappa \pm 0.038$
FP	15	169	11	82	avg:	0.84	0.62	0.98	0.68	D=15 FPP

^a^Abbreviations: precision or user’s accuracy (UA), recall or producer’s accuracy (PA), accuracy (ACC), *F*-measure, overall accuracy (*Acc*), Cohen’s kappa (*κ*) and its 95% confidence interval ($\kappa_{95}$), false positives (FP), false negatives (FN), features per pixel (FPP), dimensionality (*D*).

Results in Table 10 include accuracy assessment matrices, per class metrics, and aggregate performance measures. Matrices of all classification experiments were statistically tested against the solution provided by chance and against each other, which confirmed that they are unique and suitable for further comparisons.

**Fig 8:**
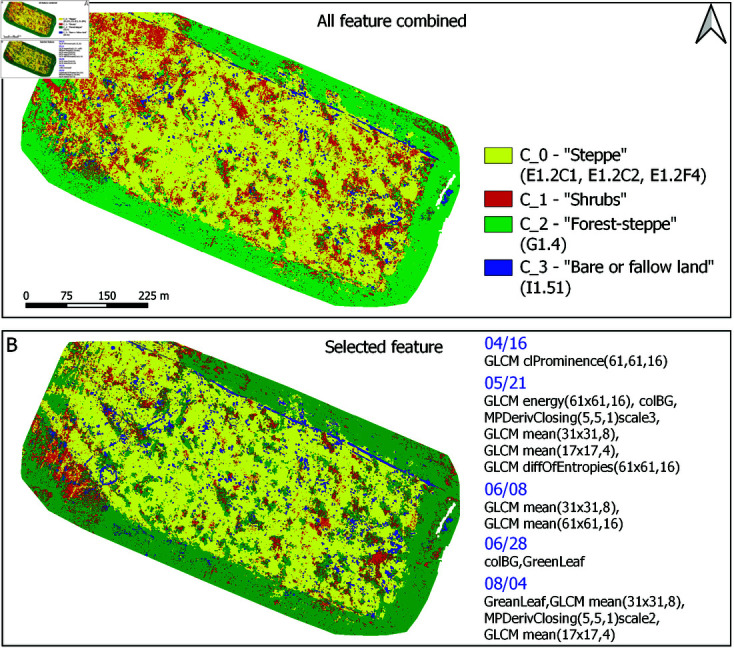
Visual comparison of image classification results. A: Map produced by the model combining all textural, morphological and spectral descriptors over collected UAV image time series (in total 444 combined features per pixel). B: Improved map produced by the model based on only 15 out of 444 features (list of features selected by the VSURF procedure is shown on the right-hand side, please see the Methods section).

Visual comparison of the two best performing classifiers in Table 10 is given in Fig 8, where the corresponding habitat type maps based on UAV time series are presented.

In comparison to all possible habitat succession phases that were defined as the result of extensive fieldwork at the test site, Table 7, image classification was focused only on the most distinctive or dominant habitat types in the test site, categories identified in Table 9 (column four).

In addition, in UAV mapping experiments in Fig 8 we redefined recognition classes by combining some of the habitat types from Table 9 into same classification categories, due to the reasons discussed in the Methods section.

Discriminative power of considered image descriptors was analyzed by using them individually (models denoted as *spectral*, *texture*, and *morphological* in Table 10), and *combined* (by performing their fusion at the feature level). In all cases classification experiments involved complete UAV image time series, since classification models based only on individual UAV images were not able to achieve acceptable performance.

The last model in Table 10 has an additional feature selection that was made in order to further improve the overall performance and reduce the number of predictor variables (ease model deployment). Feature selection was motivated by the relatively large dimensionality of *combined* feature vectors, which included time series of texture, morphological and spectral descriptors (in total 444 FPP). On the other hand, after performed feature selection only the 15 most important values were kept, Fig 8.

**Fig 9:**
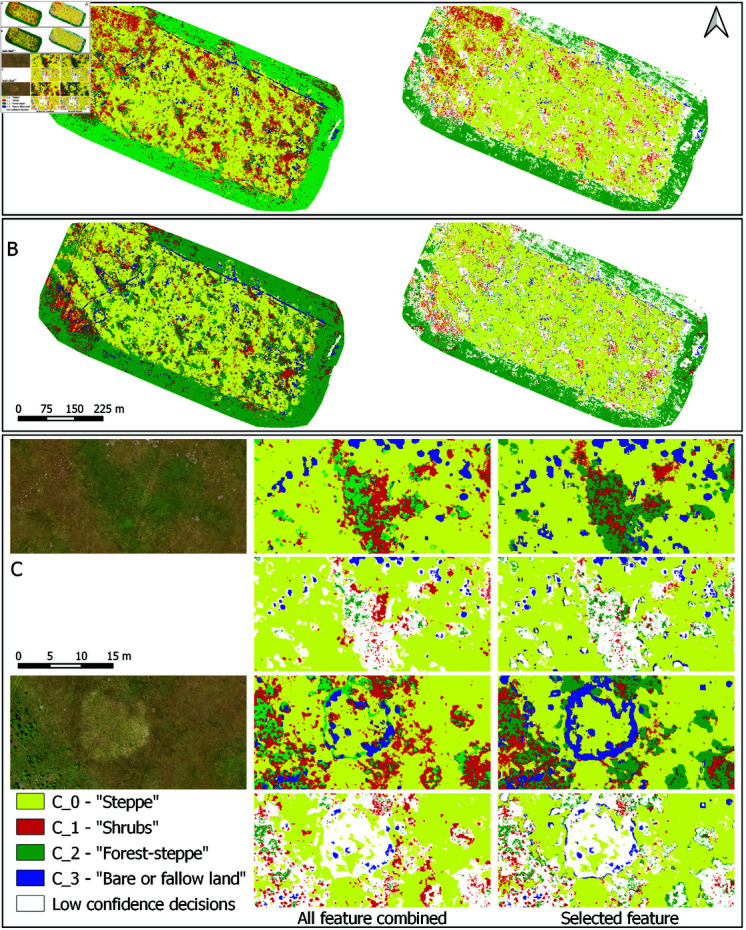
Classifier decisions with overlaid confidence scores. Pixels with low confidence scores ( < 0.6) are shown in white. A: Map produced by the model based on combined image descriptors, without feature selection, and B: Model trained on selected 15 features. C: Side-by-side comparisons for the image details shown by the two true colour images on the left.

Influence of feature selection on model performance was also assessed through confidence provided by classification scores, Fig 9. It shows results produced by the models in Table 10 trained on all 444 features, and only 15 selected image descriptors.

### Habitat extent

Produced UAV vegetation maps demonstrate that the estimated extent of steppe habitat, class *C_0_* in Fig 8 dominates identified observation Zones I, II, and V in Fig 4.

In Zones I, II, and V the steppe *C_0_* covers between 60.04 and 68.44 percent (for the proposed model with all features combined) and 58.24 and 68.43 percent (using only selected 15 features). The extent of shrubs *C_1_* ranges from 3.38 to 4.47 percent against the 1.45 to 2.28 percent, while the extent of bare soil *C_3_* ranges from 0.25 to 2.05 percent, and 0.92 to 1.90 percent, respectively. The percent of classified pixels with low confidence score across these three Zones is between 24.36 to 32.07 percent, and 24.29 to 32.56 percent after performed feature selection.

The extent of Zone III characterizes the presence of steppe cover *C_0_* dominated by shrubs *C_1_*. According to model relying on all features combined, the steppe habitat *C_0_* occupies 47.77 % of the area, followed by shrubs *C_1_* with a presence of 18.10 %. Classifier using only 15 selected features shows that the steppe *C_0_* covers 58.88 % and shrubs *C_1_* cover 3.44 % of the area. According to both classifiers, the number of classified pixels with low decision confidence scores (probability of correct decision less than 0.6) is around 31 % across Zone III.

The extent of Zone IV characterizes the presence of forest *C_2_* area range of approximately 60 percent and has around 31 percent of classified pixels with low confidence score, according to both classifiers in Fig 8.

## Discussion

### Potential of conducted UAV mapping

Analysis of aggregate performance measures in Table 10 indicates that although the most of the image classification models have relatively high overall accuracy, their characteristics are significantly different. More objective model comparison based on *κ* statistics reveals that the models based on RGB spectral features and GLCM textures are better than the ones based exclusively on pixel’s morphological profiles.

Although the RGB scenes corresponding to “Steppe” were captured at 4 cm GSD, they did not contain any specific object shapes or boundaries that would allow visual resolving of individual parts of the plants, Fig 4. Therefore, the plant mixtures of interest in the acquired grassland UAV images were mostly characterized by texture, and not by individual plant shapes (edges and their geometry).

Similarly, acquired 3-channel images did not provide a lot of spectral information, however their time series were a good indicator of the phenological changes characterized by plant colour. On the other hand, the improved results of the last two classifiers in Table 10 (denoted as combined descriptors, and combined descriptors after feature selection) confirmed that the spatial context of the pixel’s neighbourhood was able to capture necessary information about the grassland vegetation state.

Multiscale properties of the proposed GLCM texture features proved to be good for describing spatial variations that are characteristic for some of the categories and their local spatial extent, e.g. as seen in Fig 7. Similar also holds for the morphological profiles, which were shown to match the content granularity in the scene, Fig 7. Term multiscale in the case of both types of spatial features refers to the size of analysis window, or filter size.

The class *C_0_* was always the best performing among all models, due to significantly larger number of samples (imbalanced dataset), which forced the decision bias towards its direction in the feature space. The reported per class accuracy was also influenced by unbalanced number of samples, which resulted in overly optimistic individual accuracy. However, the more complex measures, like the *F* values, hopefully revealed the real per class performance and again spoke in favour of the models utilizing all types of descriptors. This also holds for their variant after applied VSURF feature selection.

Regarding the feature dimensionality, denoted by *D*, higher dimensional models are known to be suspectable to overfitting [64], especially in the considered setting with relatively small number of GT samples. This was visually observed in Fig 8A, where the spatial extent of class *C_1_* or “Shrubs” was overestimated.

After feature selection, a more robust model was designed, Fig 8B. Besides significant speed up of the model inference, by reducing the feature space dimensionality from 444 to 15, it also produced much better classification map, as shown by side by side visual comparisons in Fig 9. E.g. in Fig 9A pixels that were classified as “Shrubs” were mostly in the low confidence regions (surrounded by the white labels), while the same pixels in Fig 9B were more correctly classified as low confidence decisions, or as “Steppe” which actually was the dominant type in the surrounding area. This can also be seen in Fig 9C. Thus, the proposed feature selection lead to a more robust model, although quantitatively it produced a slightly lower cross-validation performance in Table 10. This also enabled to more correctly discard the pixels with the low confidence decision scores. It is also interesting to note that 15 selected features, listed in Fig 8B, indicate that no features from the UAV image recorded on 09/30/2021, Fig 3, were included in the classification model. This is not surprising, since it is an image from the end of September, when the grassland vegetation season is coming to an end. As already said, such classifier also has a much lower computational cost in the deployment phase.

When it comes to generalization of the proposed solution to similar mapping scenarios, conducted experiments provide valuable design insights. First of all, it is a different kind of classification problem in comparison to the one that would detect individual plants in the image, or the one that would tackle the problem by multispectral measurements. Regarding the coverage, satellite imaging with higher spatial resolution would be much easier than UAV campaigns, however, such images (with resolution less than 1 m) are usually only commercially available, which makes them unavailable to most land managers. On the other hand, UAV platforms with RGB cameras can be considered as more ubiquitously present in such cases [92,93]. Several studies have also confirmed the usefulness of UAVs compared to satellite imagery [94,95], especially for species- and site-specific cases [96]. Obtained results confirmed that the model can provide spatial extent of habitat types at the test site, but the local data could also be a type of limitation [97] for direct model usage in other areas. If the plant species at nearby locations have the same composition, it would allow for the application of the same classification model under assumption that the UAV acquisitions were taken at the approximately same times over the temporal observation window. However, even in the cases when additional retraining of the classification model would be needed, e.g. due to different habitat type categories, the proposed methodology and the results would still serve as a clear guidance for development of the mapping procedures of the same type.

Some of identified habitat types had significant variation in visual appearance over the growing season. To some extent this was expected, and has been illustrated by seasonality effect in Fig 3. This valuable information about the vegetation’s temporal dynamics was taken into account by fusion of UAV image descriptors over time series. Nevertheless, we have also investigated the potential of individual RGB images, i.e. time samples. However, obtained results were not satisfactory in terms of classification performance. Namely, the low spectral resolution and inability to visually resolve specific spatial features of habitat types severely limited discrimination between the categories. Therefore, classifications relying on the whole image time series were selected as the only appropriate ones in the given setting. On the other hand, the proposed feature selection effectively resolved the potential problem of increased model complexity.

DL methods and data driven feature engineering have been the dominant paradigm for supervised image classification in recent years. As mentioned in the Introduction, it also holds for grassland monitoring applications [44–48], where explicit object detection DL techniques can be applied if image resolution allows for identification of individual plant parts [44,45]. When resolution is lower, DL can be used for advanced classification of small image patches [47,48] and spectral data cubes [46]. However, by being completely data-driven, these patch classification DL approaches usually require large number of labeled training samples. In that sense, in the given case it was not possible to pursue a DL based classification approach, nor the data-driven feature engineering, preferably due to small number of GT samples. As pointed out by [68] or [98], it would be possible to characterize the pixel’s spatial neighbourhood by feature maps produced by an independently trained DL model. However, such texture representation was not utilized in this study due to relatively small size of the corresponding spatial analysis window around each image pixel. Namely, the input size of pretrained convolutional neural networks is usually significantly larger than the 25x25 pixels, which was the size of vegetation sampling plots in the given case of 4 cm GSD. In addition, there were only 24 vegetation sampling plots, which severely limits the number of small image patches that could be used for training or adaptation of DL models based on the inputs in the form of small image patches.

When it comes to the methods based on classification of small image patches, we would also like to point out that in the relatively recent study [99] it was clearly shown that object-based methods for grassland classification can be applied to RGB images in cases when the level of fine-scale spatial details is sufficiently high, e.g. at the GSD of the order of 2 mm. However, at the GSD of 2 cm objects in the grassland images are hard to define and multispectral information was used to compensate for the lack of spatial details [99].

Depending on available resources (camera type, mission duration/UAV flight height, and the number of ground-truth samples) the proposed design procedure could be adapted to different scenarios. Thus, although the proposed method belongs to the category of pixel-based solutions, the same research methodology could be also applied to object-based methods and ease the design of similar grassland mapping solutions.

Although the UAV images were of the relatively high resolution, 4 cm GSD (Fig 4), automated discrimination of the considered complex plant mixtures in RGB images still proved to be a challenging task The general findings of our study were also that the potential inconveniences coming from an inadequate level of spatial details, low spectral resolution, limited number of temporal observations and ground-truth samples could be avoided to certain extent by an appropriate characterization of the mapping problem.

In the given case, since the scene is non-stationary, solutions based on object-based approach would also mean that an additional problem of temporal feature fusion over defined image objects (small image patches obtained by image oversegmentation) would arise. This was one of additional reasons for pixel-based approach, since the temporal information proved to be very important for classification of grassland vegetation types.

In terms of possible improvements, higher spectral resolutions instead of RGB images would certainly ease the classification problem, and probably enable solitary UAV acquisitions instead of multitemporal ones. In terms of data sampling, we note that the number of vegetation plots used for the design of classification model was relatively low due to the amount of work that was required to continuously monitor these survey plots throughout the vegetation season by in-situ field campaigns. In that sense, studies like [100] could suggest how to efficiently design similar remote sensing studies with minimal field data in the future.

### Steppe habitat renewal and biodiversity conservation aspects

Compared to large-scale habitat-type classification, like the one described in [101], this study’s characterization of the sandy steppe habitat required a detailed analysis of vegetation types in the field, i.e., in-situ vegetation surveys that were the basis for habitat type classification. The research shows that the sandy steppe regeneration at Sunčani salaš occurs in four successive habitat development phases. The first phase lasts for a minimum of two years, the second phase a minimum of five years, and the third phase can last for five to ten years. Finally, the fourth phase lasts two years if the open habitat is not maintained by grazing or mowing operations. In that case, the habitat may get overgrown by hawthorn (*Crataegus monogyna*) and blackberry (*Rubus sp.*), and vegetation succession will move towards Forest–Steppe vegetation development. The development stage of Forest–Steppe I can last from five to up to ten years, after which the Forest–Steppe II phase can last between 10 and 20 years [102,103]. Based on data presented in Table 9, the open grassland of Sunčani salaš cannot be described as fully revitalized. [104] suggest that, whether sites are actively restored or left to return to their (somewhat) natural state, they take approximately ten years to develop towards semi-natural grasslands. Based on the results presented in Table 9, we consider 60 % of the open habitat to be characterized by young steppe vegetation (succession phases II and III), which, after 12 years is in accordance with the statement given previously. According to the results of the UAV mapping, the extent of the young steppe is the largest in zones I, II and V, where it reaches almost 70 %.

For the revitalization process to be completed, we believe that around 60 % of the area needs to be in succession phase IV. For this target to be met, appropriate management measures are needed. Intermittent grazing, as a management measure is present at Sunčani salaš since 2018 and we hope to establish a scheduled sheep grazing regime soon. At the moment, the most important threat to the revitalization process stems from the spread of shrubs and invasive species, namely common milkweed (*Asclepias syriaca*). *A. syriaca* is infamous for invading psammophilous sites with altered natural vegetation [105,106], especially in places of soil disturbance [107]. Similarly, at Sunčani salaš, plots dominated by *A. syriaca* were located near a dirt road and in places of wildlife usurpation on the southern edge of the open grassland. The species is widespread in similar habitats across Czech Republic, Romania and Poland [108,109]. Furthermore, just on the other side of the border, the same species poses a threat to sandy steppes within the Kiskunság National Park (Hungary) [105]. As already mentioned, Pannonic loess steppe grassland and Pannonic sandy steppes are recognized as habitats of conservation importance and are listed on the Annex I of the Habitats Directive [30]. Therefore, it is of the utmost significance for the protected area managers to focus measures onto areas under pressure from invasive species and shrubs. This will help not only native vegetation, but the European ground squirrel, because even though the species shows no preference to any specific plant communities when selecting its habitats [110,111], the availability of food resources plays an important role in sustaining its populations, especially in reinforced/reintroduced ones [112]. Species found to be preferred over others in EGS diet: *Achillea millefollium, Scabiosa sp., Trifolium sp., Medicago sp., Vicia sp., Thymus sp., Potentilla sp.,Veronica sp.* [34,35] are the most abundant in the central part of the site, S2 Table. The same area is also characterized by the lowest presence of bare ground and fallow land, and has the “most steppic” character, Fig 4, and is, thus, the most suitable for the life of the EGS. The detrimental role of dietary resources in population viability has been proven in ground squirrels on numerous occasions [113–117] and should not be overlooked in management plans.

Finally, it’s important to note that for the long-term viability and EGS repopulation of the entire grassland area of not just Subotica sands, but northern Vojvodina in general, grassland renewal projects of a larger scale are needed. In the current setting, small grassland patches in this area are scattered among large uniform areas used for agriculture. The connectivity of such patches on a larger, landscape-scale, is highly reduced. This, naturally, impacts biodiversity as a whole making it harder for species to disperse from one remaining patch to another [118], and species such as the EGS, with limited mobility [31,56], especially. Thus, the large-scale grassland renewal should be the way to go to improve the state of biodiversity in areas that are under high anthropogenic influence, such as Vojvodina.

## Conclusions

This study aimed to provide an efficient, cost-effective way of grassland habitat assessment for land managers. The methodology comprised of an in-situ vegetation study, the results of which were used to generate UAV images of the site and develop an automated classification of the present habitat types. The in-situ study recognized habitat types of conservation priority and located areas in need of management, taking into account the needs of the European ground squirrel, a grassland specialist recently reintroduced to the site. The proposed image classification solution was designed based on the less computationally demanding features, and even though it did not fully distinguish the fine-scale grassland heterogeneity, it proved useful in measuring the extent of the targeted habitat types and their compositional carriers. Several improvement opportunities were identified: mainly regarding the size of the sampling plots and the use of image sensors with higher spectral resolution that could bring substantial performance improvements. Although quantitative evaluations of the obtained classification models confirm their applicability, these could be further improved by incorporating plant multispectral features and employing UAV images with higher spatial resolution (which would reveal geometries of individual plants in the mixtures).

## Supporting information

S1 Table Detailed list of identified plant species on the test site. Vegetation data gathered during fieldwork campaigns, presented in tabular form, **.xlsx** file.
(XLSX)

S2 Table Summary of collected data from each sampling plot. Fieldwork data in tabular form, **.xlsx** file. Data are presented jointly in the sheet named “Plot data”, as well as in separate sheets corresponding to different sampling dates. Each of defined sampling plots was observed several times during the season, as specified in Table S2. Based on multiple observations and prevailing mixture of plant species inside the plot during the whole season, each of the plots was finally categorized into one of defined habitat type categories (Table 7). Plot numbers in S2 Table correspond to the ones in S1 Fig.
(XLSX)

S1 Fig Positions and identifiers of vegetation sampling plots. 1m x 1m plots are numbered according to “Plot No.” in Table S2, and each of plots colors corresponds to one of the habitat types at the Sunčani salaš test site (color legend as in Fig 4).
(TIF)
