# Supplementary figures and images for: High resolution descriptors for UAV mapping in biodiversity conservation – A case study of sandy steppe habitat renewal
Source: PLoS One. 2025 Mar 13;20(3):e0315399. doi: 10.1371/journal.pone.0315399 (PMC11906168; doi:10.1371/journal.pone.0315399)

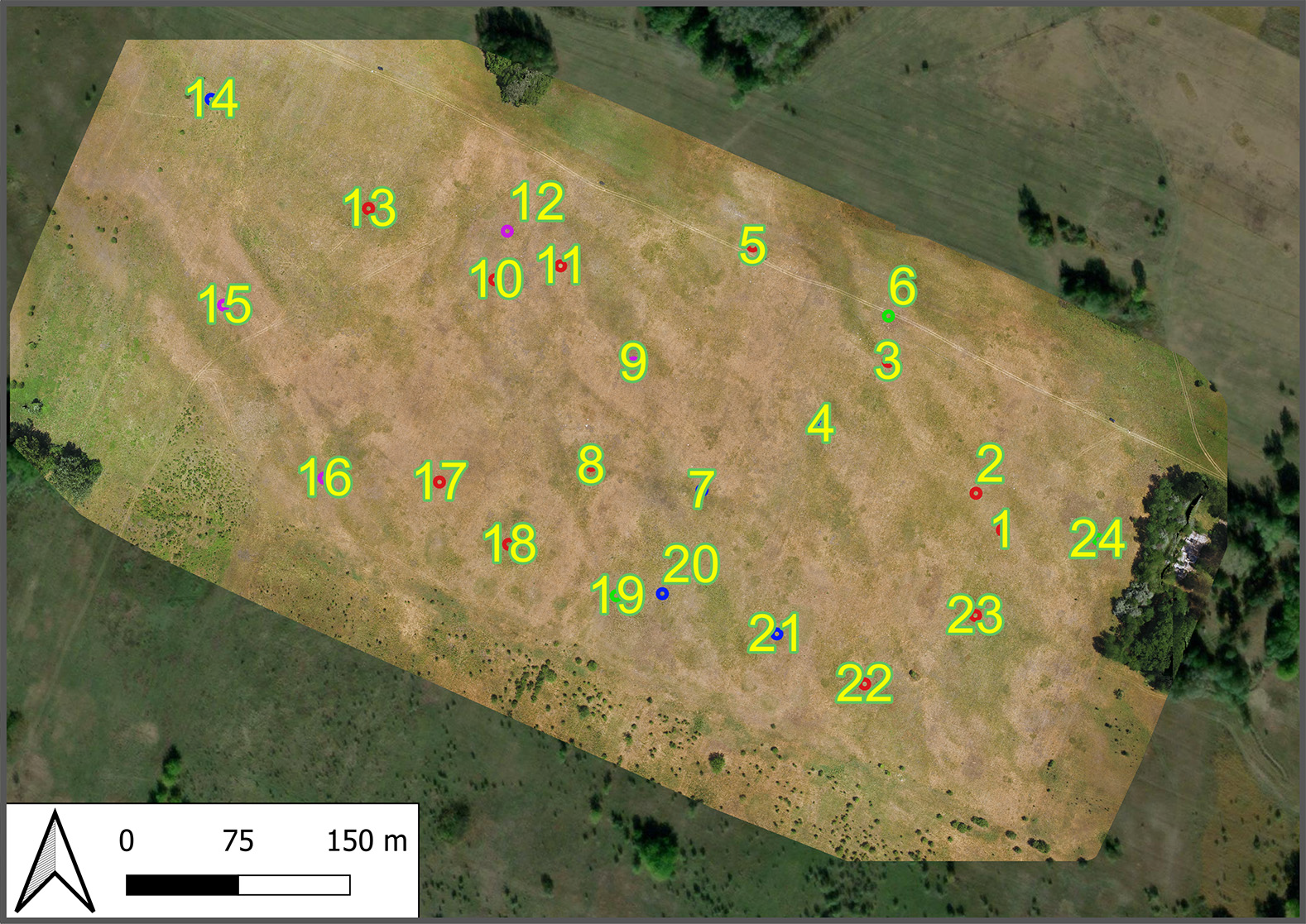

Supplement: S1 Fig — 1m x 1m plots are numbered according to “Plot No.” in Table S2, and each of plots colors corresponds to one of the habitat types at the Sunčani salaš test site (color legend as in Fig 4). (TIF) [file pone.0315399.s003.tif]
